# Minimal Increase in Total Hip Arthroplasty Surgical Procedural Time with the Use of a Novel Surgical Navigation Tool

**DOI:** 10.2174/1874325001812010389

**Published:** 2018-09-28

**Authors:** Alexander Christ, Danielle Ponzio, Michael Pitta, Kaitlin Carroll, Jeffrey M. Muir, Peter K. Sculco

**Affiliations:** 1 Division of Adult Reconstruction and Joint Replacement, Department of Orthopedic Surgery, Hospital for Special Surgery, 535 East 70th Street, New York, NY, 10021, USA; 2 Intellijoint Surgical, 60 Bathurst St., Suite 6, Waterloo, ON, N2V 2A9, Canada

**Keywords:** Total hip arthroplasty, Computer-assisted navigation, Procedural time

## Abstract

**Background:**

Computer-assisted navigation has proven effective at improving the accuracy of component placement during Total Hip Arthroplasty (THA); however, the material costs, line-of-site issues and potential for significant time increases have limited their widespread use.

**Objective:**

The purpose of this study was to investigate the impact of an imageless navigation device on surgical time, when compared with standard mechanical guides.

**Methods:**

We retrospectively reviewed prospectively collected data from 61 consecutive primary unilateral THA cases (posterior approach) performed by a single surgeon. Procedural time (incision to closure) for THA performed with (intervention) or without (control) a computer-assisted navigation system was compared. In the intervention group, the additional time associated with the use of the device was recorded. Mean times were compared using independent samples t-tests with statistical significance set *a priori* at p<0.05.

**Results:**

There was no statistically significant difference between procedural time in the intervention and control groups (102.3±28.3 mins vs. 99.1±14.7 mins, p=0.60). The installation and use of the navigation device accounted for an average of 2.9 mins (SD: 1.6) per procedure, of which device-related setup performed prior to skin incision accounted for 1.1 mins (SD: 1.1) and intra-operative tasks accounted for 1.6 mins (SD: 1.2).

**Conclusion:**

In this series of 61 consecutive THAs performed by a single surgeon, the set-up and hands-on utilization of a novel surgical navigation tool required an additional 2.9 minutes per case. We suggest that the intraoperative benefits of this novel computer-assisted navigation platform outweigh the minimal operative time spent using this technology.

## 1 INTRODUCTION

Accurate acetabular component placement during Total Hip Arthroplasty (THA) is essential to minimize bearing wear, noise generation, and post-operative complications such as instability and iliopsoas impingement [1-3]. Instability, increased wear and persistent pain secondary to component malposition may lead to decreased patient satisfaction, readmission, reoperation and an increased financial burden on the healthcare system [2-4]. As the number of primary THAs continues to increase around the world, health care systems are implementing standard of care perioperative protocols to minimize complications leading to revision surgery. The cost of revision arthroplasty is high and ranges from $29,000 to $54,000 per procedure in the United States [4, 5] and any increase in the number of preventable revision surgeries places even further economic burden on an already strained healthcare system. In addition, in the United States, the implementation of bundled payment systems and the subsequent potential penalties for hospitals and health care systems has the potential to reduce costs, with the hospital responsible for the financial penalty for 90 days postoperatively [6]. As the majority of dislocations (60%) occur during the first 3 months, the importance of maximizing accuracy when implanting THA components cannot be underemphasized [7].

Traditionally, surgeons rely on experience, anatomic landmarks, and alignment guides to guide component placement during THA. Simple mechanical alignment guides may provide additional information on cup position but are often dependant on accurate patient positioning which has been shown to change up to 20 degrees during the surgical approach and retractor placement [8]. Patient anatomic landmarks can often be obscured secondary to large acetabular osteophytes that may also obscure the transverse acetabular ligament. Since, their introduction over a decade ago, multiple studies have demonstrated the improved accuracy and precision of computer-assisted navigation devices acetabular component placement [9-12]. However, there are several drawbacks with computer navigation, including increased procedural cost, cumbersome technology, line-of-site issues and additional operative time, which have limited its widespread adoption [13]. While usage has increased over the last 5 years, most estimates suggest that navigation is used in only 5-7% of THA procedures [13, 14]. Increased operative time significantly increases overall procedural cost, which has been estimated at between $37-62 per minute in the United States [15, 16]. In addition, increased operative time reduces the total number of cases that can be complete each day, while also increasing the risk of perioperative complications, including infection [17, 18]. For this reason, any additional operative time must be recorded and justified. In several studies, traditional navigation has been shown to lengthen procedural time between 12 to 23 minutes [17-19], an increase that could potentially decrease operating room efficiency and surgeon/hospital surgical volume.

At our institution, we have begun to utilize a novel, 3D mini-optical navigation device comprised of a miniature camera and optical tracker during THA procedures. This device has demonstrated excellent accuracy [20-24] and, in early clinical studies, was not associated with a significant increase in procedural time [21]. The purpose of this study was to evaluate the impact of this novel mini-navigation system on a consecutive series of THA procedures. Our hypothesis is that there would be no significant difference in procedural time between THA cases performed with the navigation device compared to THA performed using traditional mechanical guides.

## 2 MATERIALS & METHODS

### 2.1 Study Design and Study Groups

This study was an Institutional Review Board-approved retrospective cohort study of patients who underwent primary unilateral THA *via* the posterior approach between May 2016 and June 2017 by a single surgeon.

Patients who underwent a primary unilateral posterior approach THA performed by the senior author (PKS) were included in this study. Two groups were compared: in Group 1 (control), THA was performed using standard mechanical guides and in Group 2 (intervention), THA was performed using a novel computer-assisted navigation system (Intellijoint HIP, Intellijoint Surgical, Waterloo, ON).

Primary outcomes for this study included total procedural time and time required to complete device-related tasks. Procedure time was defined as the time between primary incision and procedure completion. Device-related times were collected for each step required to use the navigation system, including: 1) installation of the pelvic platform, 2) camera aiming and registration, 3) femoral platform installation, 4) baseline leg length and hip center of rotation measurement and 5) final hip center of rotation measurement. A minimum of 5 seconds was required to be spent on a given task for that time to be recorded. Finally, total procedural time – including pre-incision tasks required for use of the navigation device – was calculated.

### 2.2 Hip Navigation System

The use of the novel imageless hip navigation tool (Intellijoint HIP, Intellijoint Surgical, Waterloo, ON) has been described in detail elsewhere previously [20, 25]. The navigation tool consists of a camera, a tracker and a computer workstation Fig. (**1**). The camera is magnetically attached to a pelvic platform, which is itself rigidly fixed to the pelvis *via* two threaded pins that are drilled into the iliac crest. The camera captures the movements and position of the tracker, which can be magnetically fixed to various instruments during surgery. Acetabular cup position is measured with the tracker attached to the impactor, while changes in leg length are measured by attaching the tracker to a platform fixed to the greater trochanter by a single screw. The tracker can also be magnetically attached to a surgical probe, thus allowing specific measurement of objects or distances. All data is relayed from the camera and captured on a laptop workstation.

### 2.3 Statistics

Statistical significance was set *a priori* at *p*<0.05 for all comparisons. Mean values were compared using independent samples t-test and/or single-factor ANOVA. Mean values are presented as mean (Standard Deviation (SD), range).

## 3 RESULTS

A total of 61 patients (30 intervention, 31 control) were evaluated for inclusion in this study. Four patients were excluded from the intervention group: one required reinstallation of the femoral platform during their procedure and three required removal of pre-existing hardware. No patients were excluded from the control group. As such, 57 patients (26 intervention, 31 control) were included in the final analysis.

### 3.1 Procedural Time

There was no statistically significant difference between procedural time in the intervention (102.3 ± 27.7 mins) and control groups (99.1 ± 14.7 mins, p = 0.60).

### 3.2 Device-Related Time

The mean total time required for all device-related tasks (both pre- and intraoperative) was 2.9 ± 1.6 mins Table **1**. Pre-incision tasks included the installation of the pelvic platform and accounted for a mean of 1.1 ± 1.1 mins. Device-specific tasks occurring during surgery accounted for 1.6 ± 1.2 mins, including installation of the femoral platform prior to dislocation, which accounted for a mean of 0.5 ± 0.5 minutes.

## 4 DISCUSSION

Accurate restoration of leg length, offset, and acetabular component positioning during THA minimizes complication and encourages optimal clinical outcomes. Computer-assisted navigation systems have been shown to improve both precision and accuracy in component placement; however, the majority of surgeons continue to rely on traditional methods and mechanical guides. Navigation systems have failed to gain widespread adoption in THA due to several drawbacks including increased cost, technical challenges, and overall longer operative times. We evaluated a novel image-less navigation system for THA and compared the procedural time of THA performed with device with that of THA performed *via* traditional methods, and found that the use of navigation did not significantly lengthen procedural time (mean 2.9 minutes). Our data suggest that this new navigation system offers a computer-assisted navigation option that provides the accuracy benefits of navigation without significantly increasing surgical time.

Current computer-assisted navigation systems provide important intraoperative data that helps to improve component selection and placement. This improved accuracy is thought to be associated with a decrease in the likelihood of dislocation and revision surgery [26, 27], and has been associated with good outcomes [28]. Indeed, several systematic reviews and meta-analyses have demonstrated improvements in component accuracy and outcomes when comparing navigation-assisted THA with traditional THA. One recent review [29] pooled results from seven randomized, controlled trials and found that the use of navigation was associated with significant improvements in the accuracy of acetabular cup orientation (anteversion: Z=3.07, p=0.002; inclination: Z=2.56, p=0.01). Several other systematic reviews have demonstrated the additional ability of navigation to limit safe zone outliers [28, 30-32], a key to the purported value of navigation in improving long-term outcomes and decreasing dislocation rates. The navigation system used in this study has itself been the subject of several recent studies examining its accuracy in comparison with post-operative imaging. Specifically, in two recent clinical studies, the device demonstrated the ability to measure anteversion to within 2.97°±4.05° [33] and 3.26°±3.11° [34] and inclination to within 1.06°±0.94° [34] and 2.17°±2.50° [33] of EOS-derived values. In another study [35], use of the device was associated with an increased proportion of acetabular components placed in the safe zone (92% *vs*. 67% traditional, p = 0.002) and a decrease in the number of post-operative leg length differentials greater than 5 mm (7.1% *vs*. 31% traditional, p=0.007).

Despite these observed improvements in component accuracy associated with navigation, there are several drawbacks to their use. The costs associated with these devices can be prohibitive for some facilities, with capital costs reaching $250,000 and software/maintenance accounting for tens of thousands of dollars of additional costs per year [36, 37]. Additionally, the learning curve associated with their use can require extended periods of time (up to 50 cases) before surgeons achieve proficiency [38]. Finally, while providing accurate measurements, these devices have been associated with significant increases in procedural time, ranging from 12 to 23 minutes per procedure [11, 17]. For high volume surgeons, increases in time of this magnitude can have a substantial impact on efficiency, resulting in a decrease in surgeon and hospital volume. As such, an important finding in our study was that the use of the device did not add significantly to procedural time. The addition of 1.6 minutes to procedural time (incision to completion) is not statistically significant, and does not represent additional time that would be associated with a decrease in operating room efficiency. Likewise, the pre-incision time of 1.1 minutes is also not statistically significant, and indicates that there is no appreciable addition to the preparation time prior to primary incision. Specifically, our study demonstrated that the device adds 2.9 minutes, all of which was accounted for by the device-specific tasks. No additional time was required due to the use of the device (*e.g.* intraoperative imaging, device calibration). In comparison, several studies have examined the additional time required for traditional imageless navigation, which has been found to add from between 12 to 23 minutes [9, 11, 17-19]. Extending procedural time by this amount has a substantial impact on efficiency. Using even the most conservative of cost estimates, traditional navigation can be associated with an increased cost of $851 per primary THA, compared with a more modest increase of $60 for the system used in this study.

Our study has several limitations. The retrospective nature of our study is limiting, and future prospective studies should be performed to assess a larger cohort of patients. Additionally, this study summarizes the results of one surgeon at a high-volume institution which may make the results difficult to extrapolate to lower volume and/or less experienced surgeons. However, the senior author practices at an academic institution, frequently teaching residents and fellows, which would increase overall surgical time compared to surgeons in non-academic settings.

## CONCLUSION

We found that a novel mini-navigation tool for total hip arthroplasty did not significantly lengthen procedural time while providing the precision and accuracy benefits of computer-assisted navigation.

## Figures and Tables

**Fig. (1):**
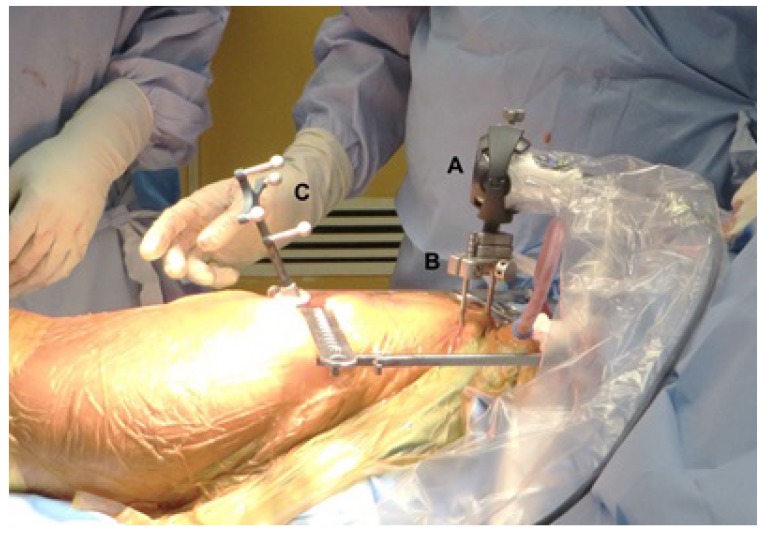
The Intellijoint HIP^®^ mini-navigation system. The camera **(A)** sits atop a pelvic platform **(B)**, fixed to the iliac crest *via* two surgical pins. The camera tracks movement of the tracker **(C)**, which can be magnetically attached to a platform fixed to the greater trochanter (as illustrated) or to other objects during surgery (*e.g.* impactor, surgical probe).

**Table 1:** Summary of time associated with computer navigation-related tasks.

Pelvic Platform Installation (mins)	Camera Aiming and Registration (mins)	Femoral Platform Installation (mins)	Baseline Leg Length/hip Center^1^ Capture (mins)	New Hip Center Capture^1^ (mins)	Total Device-related Time (mins)	Pre-incision Time (mins)
1.1 (1.1)	0.75 (0.88)	0.50 (0.53)	0.43 (0.53)	0 (0)^2^	2.9 (1.6)	1.1 (1.1)

1. Hip center of rotation. 2. Minimum time required to record was 5 seconds.
